# Editorial: Non-coding RNA and Coronary Heart Disease

**DOI:** 10.3389/fcvm.2022.910396

**Published:** 2022-05-13

**Authors:** Shu He, Laiyuan Wang, Xiangming Ding, BuChun Zhang, En-Zhi Jia

**Affiliations:** ^1^Department of Cardiovascular Medicine, The First Affiliated Hospital of Nanjing Medical University, Nanjing, China; ^2^Key Laboratory of Cardiovascular Epidemiology & Department of Epidemiology, Fuwai Hospital, National Center for Cardiovascular Diseases, Chinese Academy of Medical Sciences & Peking Union Medical College, Beijing, China; ^3^Children's Hospital Los Angeles, University of Southern California, Los Angeles, CA, United States; ^4^Department of Cardiology, The First Affiliated Hospital of USTC, Division of Life Sciences and Medicine, University of Science and Technology of China, Hefei, China

**Keywords:** non-coding RNA, MicroRNAs, lncRNAs, circRNA, coronary heart disease

## Introduction

There are only 1.5% of the human genome contains genetic sequences that encode proteins of the human genome, and more than 80% of the genomic sequences are non-coding RNAs (ncRNAs) that are not involved in the protein translation process (1). Regarding their regulatory roles, ncRNA was divided into housekeeping ncRNAs (rRNA, tRNA, snRNA, snoRNA, etc.) and regulatory ncRNAs (miRNA, siRNA, piRNA, lncRNA, circRNA, etc.) (2). Regulatory ncRNAs can function as regulators of gene expression at the epigenetic, transcriptional, and post-transcriptional levels, and miRNAs, lncRNAs, and circRNAs have been continuously investigated in-depth due to their extensive expression and numerous regulatory roles in biological processes. With the development of next-generation sequencing technologies, the research of ncRNA has been greatly advanced. There are plenty of studies that revealed the regulatory role of ncRNA in the occurrence and development of various diseases, predominantly including tumors, neurodegenerative pathologies, autoimmune diseases, cardiovascular diseases, etc. Coronary heart disease (CHD) is a heart condition in which the arteries of the heart do not deliver enough oxygen-rich blood to the heart, resulting in myocardial ischemia, hypoxia, or necrosis. Recent studies have demonstrated that lots of ncRNAs have participated in the occurrence and progression of CHD (3–5). These ncRNAs may act as a new diagnostic basis and therapeutic target for coronary heart disease.

## ncRNAs in Coronary Heart Disease

miRNAs are small fragments of only 21–23nt in length, which are stably identified in body fluids and are resistant to degradation by endogenous RNA enzymes (6). Solingen et al. identified seven novel miRNAs that decrease proprotein convertase subtilisin/kexin type 9 (PCSK9) expression through high-throughput screening of the human microRNA library. These miRNAs will down-regulating PCSK9 secretion and up-regulate hepatic low-density lipoprotein receptor surface expression, which will mark a reduction in the risk of atherosclerotic cardiovascular disease. Matshaz et al. identified 30 dysregulated miRNAs in the whole blood of 48 hypertension females by next-generation sequencing. The differently expressed miRNA was associated with pathways such as platelet activation, calcium signaling, and vascular smooth muscle contraction pathways, which are particularly important in cardiovascular pathogenesis. Moreover, miR-1299 and miR-30a-5p are validated via RT-qPCR in a larger, independent sample in a sub-Saharan population to confirm the miRNA sequencing results. Ghafouri-Fard et al. and Zhang et al. reviewed how miRNAs function in the pathophysiological process in CHD, such as modulation of angiogenesis, lipid metabolism, inflammatory responses. What's more, their diagnostic/prognostic significance as potential biomarkers are also explored.

Available evidence indicates that lncRNAs are widely involved in the pathology of cardiac development, atherosclerosis, myocardial infarction, hypertension, and aneurysms (7–9). Current studies on the involvement mechanisms of lncRNA in CHD are mainly concentrated on constructing lncRNA-miRNA-mRNA regulatory networks based on competing endogenous RNA (ceRNA) theory. Ji et al. constructed 13 ceRNA regulatory pathways and the possible biological functions regulated by these pathways include programmed cell death, ferroptosis, and pyroptosis. Bioinformatics analysis was applied to select hub genes from microarray databases, and online databases including TargetScan, miRanda, and Tarbase were used to predict miRNA-target interactions, as reported by Zheng et al. Furthermore, ncRNA in the ceRNA networks of programmed cell death-related genes was verified in an animal model which makes the consequence credible.

The circRNA, which is highly conserved with a closed circular structure, is stable and insusceptible to RNA exonuclease (10). Its close association with CHD development makes it a potential candidate for biomarkers in the diagnosis and treatment process. Gain-of-function and loss-of-function of circRNAs hold promise as therapeutic targets. The upregulated expression of circRNA ABCA1 and circRNA KHDRBS1 in atherosclerotic vessels and oxidative-stress damaged endothelial cells were detected by Li et al. In addition, the underlying microRNA and mRNA Target genes were further verified, which provided new ideas for future studies on the biological functions of circRNA in regulating CHD. The review of Min et al. summarizes the current knowledge on molecular mechanisms and clinical implications of circRNAs. They highlight the role of circRNAs as ceRNA in atherosclerosis, myocardial infarction, cardiac fibrosis heart failure, and aneurysm, which will open up new avenues for further CHD research. The clinical application of circRNAs as molecular drugs or targets is also discussed here, and they argue that this remains a huge challenge and awaits extensive investigation.

Programmed cell death includes apoptosis, necrosis, autophagy, and a novel iron-dependent form of ferroptosis (11, 12). In this topic, the researchers observed the function of ncRNA in coordinating ferroptosis during I/R myocardial injury and heart failure. Sun et al. highlight finding from the function of miR-135b-3p in exacerbating cardiomyocyte ferroptosis, providing a new therapeutic target for improving I/R injury. There are numerous mechanisms by which ncRNAs perform the function and impact the development of CHD. The investigation of novel mechanisms including ferroptosis is essential for us to explore the secrets of ncRNA.

Epigenetics is heritable changes gene expression without altering in the nucleotide sequence. Chen et al. pay their attention to DNA methylation and described two hypomethylation gene that that is closely associated with obesity. This study provides evidence of the relationship between DNA methylation and gene expression in obese patients. Matshaz et al. investigated the involvement of DNA methylation on the pathogenesis of hypertension at the same time. They will help us explore the potential relationship between DNA methylation and ncRNA expression on the regulation of CHD.

## Concluding Remarks

**Figure 1:**
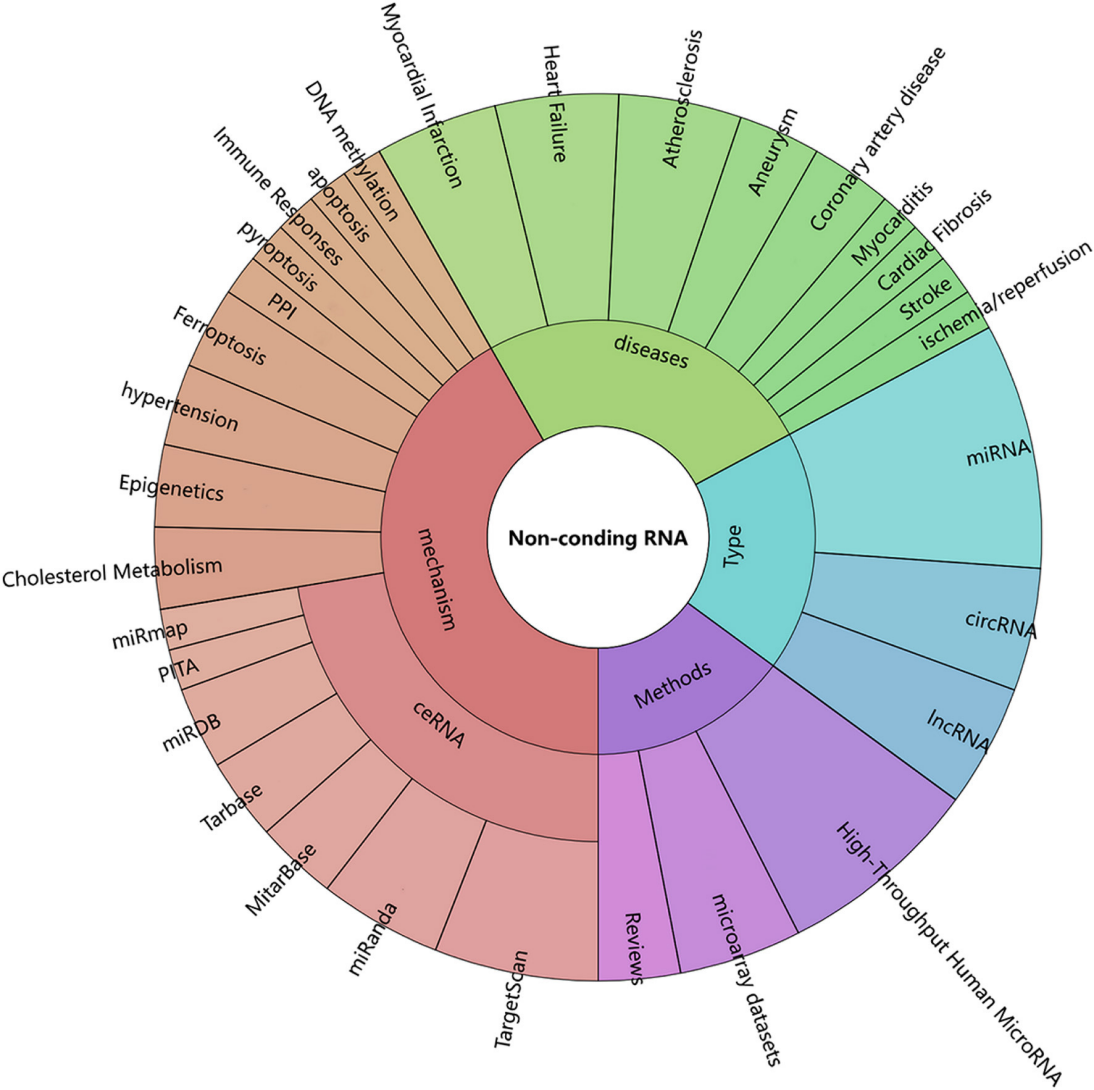
The analysis of methods and the specific mechanisms involved in the topic.

In this Research Topic, the reports address in detail the implication of ncRNA in the progress CHD. The analysis of methods and the specific mechanisms involved in these reports can be seen in Figure 1. The value of ncRNA in the diagnosis and prognosis of coronary heart disease has also been reported, while its diagnostic sensitivity and specificity remain poorly discussed. It is worth noting that the transformation from research to the clinical application will be a long-drawn-out process, and whether these ncRNAs are influenced by other factors in terms of their role in CHD needs to be verified in larger clinical trials. It is believed that in the near future, a larger amount of ncRNAs will be discovered to be involved in the development of CHD, and ncRNAs will serve as novel biomarkers and therapeutic targets for CHD.

## Author Contributions

SH and EJ were editors of this Research Topic and wrote this editorial jointly. All authors contributed to the article and approved the submitted version.

## Conflict of Interest

The authors declare that the research was conducted in the absence of any commercial or financial relationships that could be construed as a potential conflict of interest.

## Publisher's Note

All claims expressed in this article are solely those of the authors and do not necessarily represent those of their affiliated organizations, or those of the publisher, the editors and the reviewers. Any product that may be evaluated in this article, or claim that may be made by its manufacturer, is not guaranteed or endorsed by the publisher.
